# Supplementary material for: Extending the ecological model of distress to social functioning among refugees and asylum-seekers
Source: Epidemiol Psychiatr Sci. 2026 Apr 7;35:e22. doi: 10.1017/S2045796026100614 (PMC13122549; doi:10.1017/S2045796026100614)
Supplement: Kurt et al. supplementary material [file S2045796026100614sup001.docx]

**Supplementary Material Table 1**. List of conflict-related traumatic experiences

| Conflict-related traumatic experiences | N | % |
| --- | --- | --- |
| 1. Ill health without access to medical care | 729 | 59 |
| 1. Lack of food or water | 725 | 58.7 |
| 1. Lack of shelter | 652 | 52.8 |
| 1. Being close to death | 537 | 43.5 |
| 1. Serious injury | 517 | 41.9 |
| 1. Forced separation from family members | 496 | 40.2 |
| 1. Natural disaster | 452 | 36.6 |
| 1. Imprisonment | 450 | 36.4 |
| 1. Physical assault | 437 | 35.4 |
| 1. Forced isolation from others | 386 | 31.3 |
| 1. Torture | 373 | 30.2 |
| 1. Serious accident, fire, or explosion | 357 | 28.9 |
| 1. Combat situation (soldier or civilian) | 305 | 24.7 |
| 1. Unnatural death of family or friend | 299 | 24.2 |
| 1. Lost or kidnapped | 261 | 21.1 |
| 1. Brainwashing | 253 | 20.5 |
| 1. Murder of family or friend | 223 | 18.1 |
| 1. Rape or sexual abuse | 190 | 15.4 |
| 1. Murder of stranger or strangers | 176 | 14.3 |

**Supplementary Material Table 2.** List of post-displacement stressors.

| Post-displacement stressors* | N | % |
| --- | --- | --- |
| 1. Difficulties related to work | 1016 | 82.3 |
| 1. Not having enough money for daily needs | 995 | 80.6 |
| 1. Worries about visa/refugee status | 988 | 80 |
| 1. Fear of being sent back to country of origin | 984 | 79.7 |
| 1. Difficulties accessing or undertaking study | 949 | 76.8 |
| 1. Difficulties accessing physical health treatment | 865 | 70 |
| 1. Not being able to leave Indonesia | 843 | 68.3 |
| 1. Worry about safety or wellbeing of family members | 790 | 64 |
| 1. Being unable to return home in an emergency | 779 | 63.1 |
| 1. Not knowing where one will live in the future | 779 | 63.1 |
| 1. Difficulties accessing medications | 776 | 62.8 |
| 1. Loneliness or isolation | 749 | 60.6 |
| 1. Boredom | 722 | 58.5 |
| 1. Meeting financial obligations to family back home | 709 | 57.4 |
| 1. Difficulties accessing mental health treatment | 668 | 54.1 |
| 1. Worry about media portrayal of refugees/asylum seekers | 652 | 52.8 |
| 1. Separation from family | 631 | 51.1 |
| 1. Difficulties related to housing | 631 | 51.1 |
| 1. Not having access to hygiene products | 612 | 49.6 |
| 1. Difficulty accessing urgent NGO/charity support | 608 | 49.2 |
| 1. Difficulties accessing clean water | 601 | 48.7 |
| 1. Difficulties understanding immigration procedures | 588 | 47.6 |
| 1. Difficulties related to comfort (too hot/cold) | 573 | 46.4 |
| 1. Difficulties accessing or engaging in leisure activities | 566 | 45.8 |
| 1. Not having a healthy physical environment | 562 | 45.5 |
| 1. Feeling not treated as a human being | 557 | 45.1 |
| 1. Worry about safety and wellbeing of children | 540 | 43.7 |
| 1. Not having access to information in daily life | 525 | 42.5 |
| 1. Difficulties with immigration officials | 518 | 41.9 |
| 1. Difficulties caring for someone with a health condition | 505 | 40.9 |
| 1. Difficulties accessing transport | 485 | 39.3 |
| 1. Difficulties accessing communication methods | 473 | 38.3 |
| 1. Not feeling safe in your daily life | 460 | 37.2 |
| 1. Difficulties engaging in cultural practices | 441 | 35.7 |
| 1. Discrimination | 440 | 35.6 |
| 1. Difficulties communicating with other people (e.g., language barriers) | 415 | 33.6 |
| 1. Difficulties engaging in important social activities | 409 | 33.1 |
| 1. Difficulty feeling accepted by local community (racial/religious differences) | 289 | 23.4 |
| 1. Conflict with other people | 260 | 21.1 |
| 1. Difficulties with the law (e.g., police, fines) | 255 | 20.6 |
| 1. Not feeling safe in home | 242 | 19.6 |
| 1. Difficulties engaging with the police | 150 | 12.1 |

*Items are dichotomized to indicate whether each statement was rated as a moderate to serious problem versus not. Values in the table represent the number and percentage of participants who reported each item as a moderate to serious problem.

**Supplementary Table 3.** Standardized direct and indirect paths for group membership

|  |  |  | 95%CI^a^ |  |
| --- | --- | --- | --- | --- |
|  | *β* (SE) | p value | LLCI | ULCI |
| **Direct paths** |  |  |  |  |
| CTE 🡪 PDS | 0.45 (0.03) | 0.000 | 0.381 | 0.513 |
| CTE 🡪 MH | 0.14 (0.05) | 0.004 | 0.047 | 0.232 |
| CTE 🡪 GM | 0.14 (0.04) | 0.001 | 0.053 | 0.227 |
| PDS🡪 MH | 0.46 (0.05) | 0.000 | 0.372 | 0.550 |
| PDS 🡪 GM | -0.09 (0.04) | 0.037 | -0.179 | -0.009 |
| **Indirect paths** |  |  |  |  |
| CTE 🡪 PDS 🡪 MH | 0.21 (0.03) | 0.000 | 0.162 | 0.253 |
| CTE 🡪 PDS 🡪 GM | -0.42 (0.02) | 0.040 | -0.083 | -0.004 |

^a^Confidence intervals of standardized results are report. LLCI: lower-level confidence interval. ULCI: upper-level confidence interval. CTE: Conflict-related traumatic experiences. PDS: Post-displacement stressors. MH: Mental health symptoms. GM: Group membership.

**Supplementary Table 4.** Standardized direct and indirect paths for receiving support (both individuals and groups)

|  |  |  | 95%CI^a^ |  |
| --- | --- | --- | --- | --- |
|  | *β* (SE) | p value | LLCI | ULCI |
| **Direct paths** |  |  |  |  |
| CTE 🡪 PDS | 0.45 (0.03) | 0.000 |  |  |
| CTE 🡪 MH | 0.13 (0.05) | 0.004 | 0.041 | 0.222 |
| CTE 🡪 RS | 0.08 (0.04) | 0.060 | 0.000 | 0.160 |
| PDS🡪 MH | 0.46 (0.05) | 0.000 | 0.374 | 0.547 |
| PDS 🡪 RS | -0.08 (0.04) | 0.069 | -0.156 | 0.005 |
| **Indirect paths** |  |  |  |  |
| CTE 🡪 PDS 🡪 MH | 0.21 (0.02) | 0.000 | 0.162 | 0.258 |
| CTE 🡪 PDS 🡪 RS | 0.08 (0.04) | 0.060 | -0.074 | 0.002 |

^a^Confidence intervals of standardized results are report. LLCI: lower-level confidence interval. ULCI: upper-level confidence interval. CTE: Conflict-related traumatic experiences. PDS: Post-displacement stressors. MH: Mental health symptoms. RS: Receiving support from individuals and groups.
